# Catalytic Effects of Cr on Nitridation of Silicon and Formation of One-dimensional Silicon Nitride Nanostructure

**DOI:** 10.1038/srep31559

**Published:** 2016-08-16

**Authors:** Feng Liang, Lilin Lu, Liang Tian, Faliang Li, Haijun Zhang, Shaowei Zhang

**Affiliations:** 1 The State Key Laboratory of Refractories and Metallurgy, Wuhan University of Science and Technology, Wuhan 430081, China; 2 College of Engineering, Mathematics and Physical Sciences, University of Exeter, Exeter Ex4 4QF, U.K

## Abstract

The catalytic effects of chromium (Cr) on the direct nitridation of silicon (Si) and morphology of nitridation product were investigated. Cr dramatically improved the conversation of Si to silicon nitride (Si_3_N_4_). The complete conversion was achieved at 1350 °C upon addition of 1.25 wt% Cr. This temperature was much lower than that required in the case without using a catalyst. Meanwhile, Cr played an important role in the *in-situ* growth of one-dimensional (1-D) α-Si_3_N_4_ nanostructures. α-Si_3_N_4_ nanowires and nanobelts became the primary product phases when 5 wt% Cr was used as the catalyst. The growth processes of the 1-D α-Si_3_N_4_ nanostructures were governed by the vapor-solid mechanism. First-principle calculations suggest that electrons can be transferred from Cr atoms to N atoms, facilitating the Si nitridation.

Silicon nitride (Si_3_N_4_) possesses many superior properties, in particular, excellent thermal shock resistance, mechanical properties and chemical stability at both room and high temperatures^1^,^2^. Therefore, Si_3_N_4_ based materials are extensively used in a variety of areas such as in chemical reaction vessels, heat exchanger bearings, engine and gas turbines, high-temperature components, automotive parts, and aerospace vehicles^3^,^4^.

Several methodologies and techniques have been developed to produce Si_3_N_4_ powders from siliceous raw materials, including carbothermal reduction nitridation of silica^5^,^6^, chemical vapor deposition^7^,^8^,^9^, direct nitridation of silicon (Si)^10^, sol-gel processing^11^,^12^, and combustion synthesis^13^,^14^. Among these, the direct nitridation of Si powder is regarded as a low-cost and straightforward route for the large scale production of Si_3_N_4_ powder and bulk Si_3_N_4_ based materials. Unfortunately, with this technique, much unreacted Si often remains in the final products, due to partial Si melting caused by the high nitridation temperature used and additional heat released from the strong exothermic reaction between Si and nitrogen.

One of the strategies considered previously to address this issue was use of a suitable catalyst for the Si nitridation process. So far, catalytic effects of several metals on the kinetics of Si nitridation, α-/β-phase ratio and product morphology have been investigated^15^,^16^,^17^,^18^,^19^,^20^. Among the non-transition metals tested, only calcium (Ca) showed some accelerating effect on the conversion from Si to Si_3_N_4_ and the formation of α-Si_3_N_4_. On the other hand, among the transition metals, cobalt (Co) was found to show significant accelerating effects on the Si nitridation and *in-situ* growth of α-Si_3_N_4_ nanorods or nanowires^21^. Nevertheless, when it is used as a catalyst, low melting Co and/or Co-Si alloy phases will remain after the nitridation, which could potentially deteriorate high-temperature properties of the final product materials^22^,^23^. Therefore, it is necessary to use other alternative metal catalysts to overcome this problem.

One of the candidate metal catalysts for this could be chromium (Cr). Cr, chromium nitride and Cr_x_Si_y_ phases all have high melting points and good high temperature properties^24^,^25^,^26^. Therefore, their remaining after the nitridation would not have much negative effects on high temperature properties of the final product materials. However, whether Cr is catalytically active in the Si nitridation is still in controversy. Cofer and Lewis^27^ claimed that Cr could accelerate the Si nitridation via promoting the dissociative chemisorption of nitrogen. On the other hand, according to Pavarjarn *et al*.^15^, Cr actually had no obvious effect on the Si nitridation even at 1300 °C.

In the present work, the effects of Cr with various addition levels (up to 10%) on the direct nitridation of Si were investigated, and morphologies of Si_3_N_4_ products examined in detail. To assist understanding the role played by Cr in the nitridation process, first-principle calculations were also carried out. The experimental and calculated results were discussed, based on which, the relevant catalytic reaction mechanisms proposed.

## Results

### Effects of Cr additions on Si nitridation

**Figure 1:**
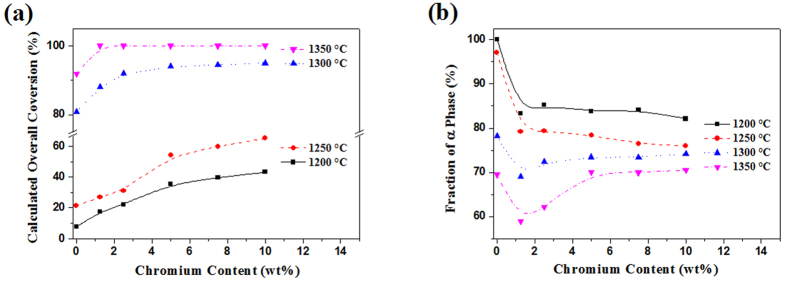
Effects of Cr content on (**a**) the overall conversion of silicon and (**b**) the formation of α-phase in samples resultant from 3 h nitridation at different temperatures.

Figure 1 illustrates the effects of Cr content on the overall conversion (OC) of Si and α-phase contents in samples resultant from 3 h nitridation at different temperatures. At 1200 °C and 1250 °C, the OC of Si to Si_3_N_4_ in the reference samples without Cr was low (Fig. 1a). However, it increased evidently with increasing the Cr content. For example, at 1250 °C, the OC was only ~21% in the case of no Cr addition, but increased significantly to ~66% in the sample containing 10 wt% Cr. Upon further increasing the temperature to 1350 °C, the OC in the reference sample increased to 91% whereas nearly all of the Si had been nitrided in the sample containing 1.25 wt% Cr. Figure 1b shows α-phase contents in the samples containing different amounts of Cr after 3 h nitridation at different temperatures. With increasing the nitridation temperature, the α-Si_3_N_4_ content decreased significantly, which was in the agreement with that reported previously^15^. The effect of Cr addition on the α-phase content appeared to be rather complicated. Overall, at low temperatures, the α-phase content decreased with the Cr addition, whereas at high temperatures, it initially decreased and then increased with the Cr addition. We think that the complicate effect of Cr on the α-phase content might be explained by the following two reasons: 1) one hand, considering the low thermal conductivity of Cr (93.7 W/mK) comparing with that of Si_3_N_4_ (~450 W/mK) and Si (150 W/mK), the excess reaction heat arisen from the nitridation of Si might not be released immediately, resulting in local overheating and α → β phase transition of Si_3_N_4_; and 2) on the other hand, the high amount of added Cr might act as diluents to absorb certain reaction heat generated from the nitridation process, then reduce the local overheating and decrease the phase transition of Si_3_N_4_.

**Figure 2:**
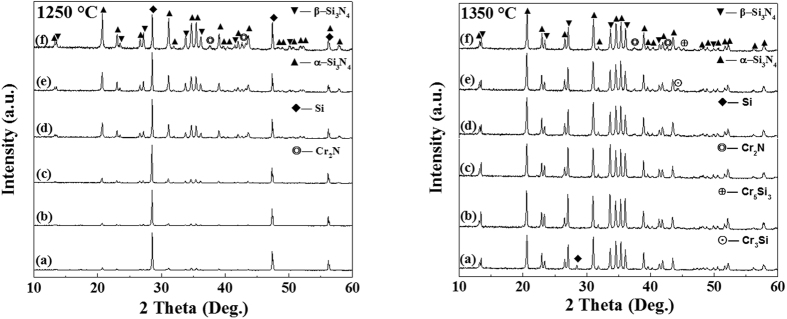
XRD patterns of samples containing various amounts of Cr: (**a**) 0, (**b**) 1.25, (**c**) 2.5, (**d**) 5, (**e**) 7.5, and (**f**) 10 wt%, after 3 h nitridation at 1250 °C and 1350 °C, respectively.

Figure 2 shows XRD patterns of samples containing 0–10% Cr after 3 h nitridation at 1250 °C and 1350 °C respectively. At 1250 °C, unreacted Si peaks remained as the main phase in the reference sample. With increasing the Cr addition, the α/β-Si_3_N_4_ peaks increased evidently whereas Si peaks decreased, indicating the great accelerating effect of Cr on the Si nitridation. Minor Cr_2_N was detected in the sample containing 10 wt% Cr. At 1350 °C, α- and β-Si_3_N_4_ were identified in the reference sample, along with some unreacted Si. On the other hand, in the sample containing 1.25 wt% Cr, Si disappeared and only α- and β-Si_3_N_4_ phases were present, indicating the complete conversion from Si to Si_3_N_4_. On increasing Cr to ≥5 wt%, α- and β-Si_3_N_4_ remained as the primary phases, however, minor Cr_2_N and two other impurity phases (appeared to be Cr_3_Si and Cr_5_Si_3_) were detected.

**Figure 3:**
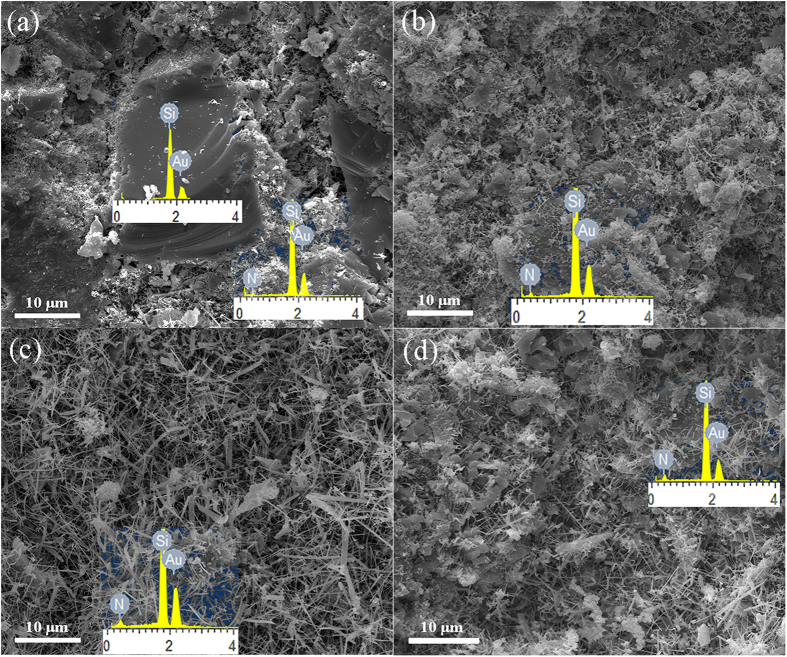
SEM images of fracture surfaces of samples containing various amounts of Cr: (**a**) 0, (**b**) 1.25, (**c**) 5%, and (**d**) 10 wt%, after 3 h nitridation at 1350 °C. Insets show EDS patterns.

Shown in Fig. 3 are SEM images of samples after 3 h nitridation at 1350 °C. Some unreacted Si was identified in the reference sample without Cr by EDS (the inset 1 in Fig. 3a), which was consistent with the XRD results shown in Fig. 2b, and a few one-dimensional (1-D) nanostructural phases were occasionally seen in the sample. EDS (the inset 2 in Fig. 3a), along with the XRD results (Fig. 2b), confirmed that the crystalline phases surrounding the unreacted Si were Si_3_N_4_, suggesting that the nitridation of Si particles proceeded from the surface towards the center. Compared to the reference sample, much less unreacted Si phases but much more Si_3_N_4_ phases were identified by EDS (the insets in Fig. 3b–d) in the samples containing Cr. Furthermore, with increasing the Cr content from 1.25 to 5 wt%, the quantity of 1-D nanophases also increased evidently.

**Figure 4:**
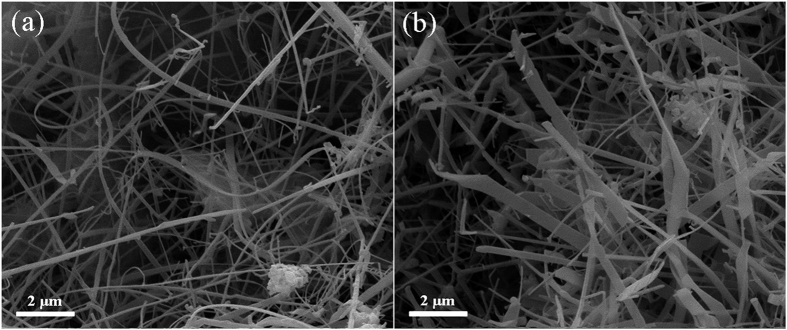
High-magnification SEM of the 1-D nanostructures formed in the sample containing 5 wt% Cr: (**a**) nanowires and (**b**) nanobelts, after 3 h nitridation at 1350 °C.

High-magnification SEM images (Fig. 4) further reveal that most of the 1-D nanophases actually possessed wire-, belt- and branched belt-like morphologies. The nanowires were 50–200 nm in diameter and about 50 μm in length, and the nanobelts were 300–1000 nm in width and about 10 μm in length. However, upon addition of >5% Cr, the quantity of 1-D nanowires/nanobelts appeared to be decreased and more Si_3_N_4_ particles coexisted with them.

**Figure 5:**
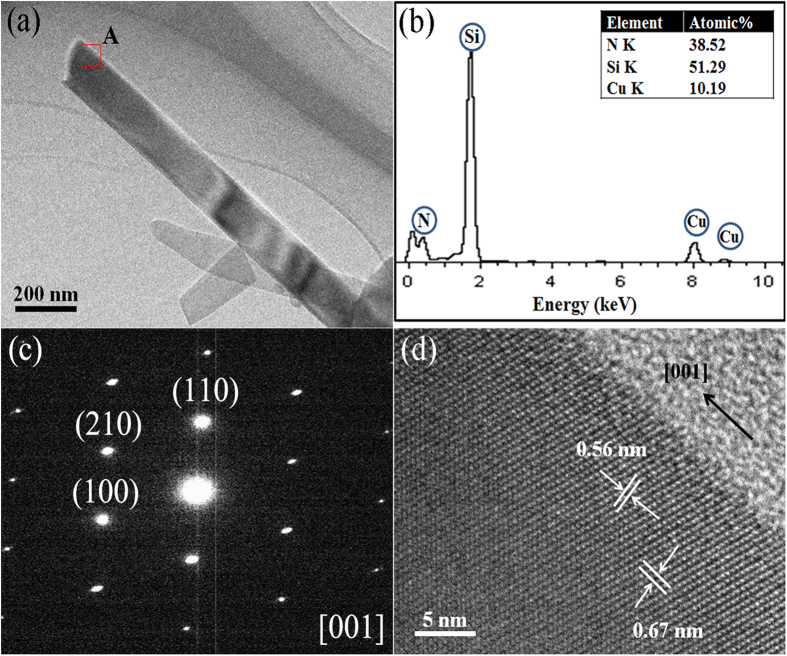
TEM images of the 1-D α-Si_3_N_4_ nanostructures in the sample containing 5 wt% Cr after 3 h nitridation at 1350 °C: (**a**) a typical low-magnification TEM image of a representative the α-Si_3_N_4_ nanowires, (**b**) corresponding EDS spectrum and (**c**) SAED pattern of the nanowire, and (**d**) an HRTEM image of the α-Si_3_N_4_ nanowire (the area A in Fig. 5a).

Figure 5 further presents TEM, HRTEM, EDS and SAED of a representative nanowire formed in the sample containing 5 wt% Cr, after 3 h nitridation at 1350 °C, showing smooth surface and uniform diameter of the nanowire (Fig. 5a). EDS (Fig. 5b) further revealed that the nanowire contained Si and N in the atomic ratio of 0.751, almost the same as the stoichiometric ratio (0.750) of Si_3_N_4_ (Cu peaks were from the copper grid sample holder), verifying that it was Si_3_N_4_. Also SAED (Fig. 5c) confirmed that is was single-crystal α-Si_3_N_4_. In addition, two-dimensional lattice fringes with d-spacing values of 0.56 nm and 0.67 (Fig. 5d) matched with the (001) and (100) planes, respectively, suggesting that the α-Si_3_N_4_ nanowire grew along the [001] direction.

**Figure 6:**
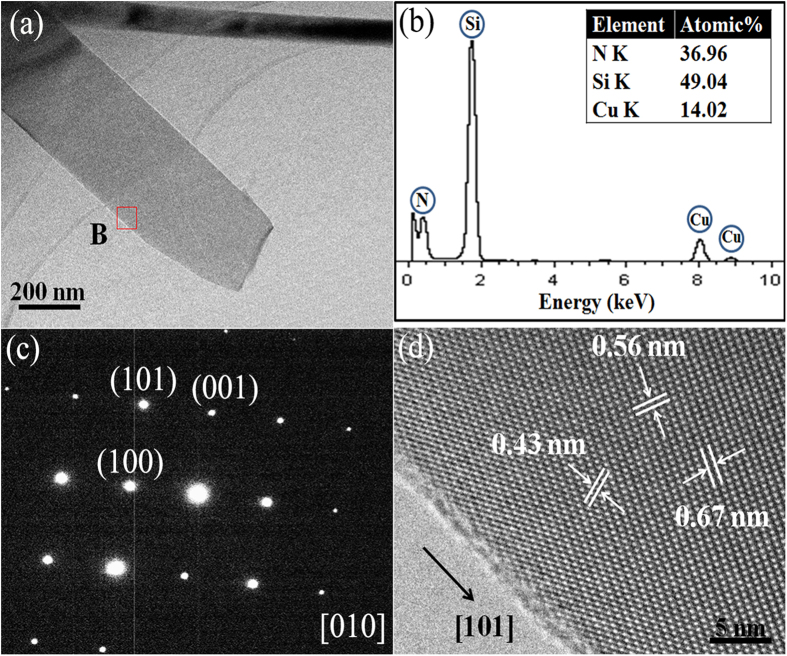
(**a**) A low-magnification TEM image of a representative α-Si_3_N_4_ nanobelt, (**b**) corresponding EDS spectrum and (**c**) SAED pattern of the nanobelt, and (**d**) a high-resolution TEM image of the α-Si_3_N_4_ nanobelt (the area B in Fig. 6a).

Apart from Si_3_N_4_ nanowires, as mentioned above, many nanobelts were also formed in the Cr added samples. As revealed by TEM (Fig. 6a), they had different widths but their widths were uniform along the entire length. EDS (Fig. 6b), SAED (Fig. 6c) and HRTEM (Fig. 6d) identified that they were also single crystal α-Si_3_N_4_ but were grown along the [101] direction. It should be pointed out that no particles were observed and no Cr was detected by EDS at tips of the 1-D Si_3_N_4_ nanowires and nanobelts (Figs 5b and 6b), suggesting that their growth processes should not have been dominated by the well-established vapor-liquid-solid (VLS) tip-growth mechanism^28^. The detailed growth mechanism regarding this will be discussed in more detail in Section 3.3 below.

**Figure 7:**
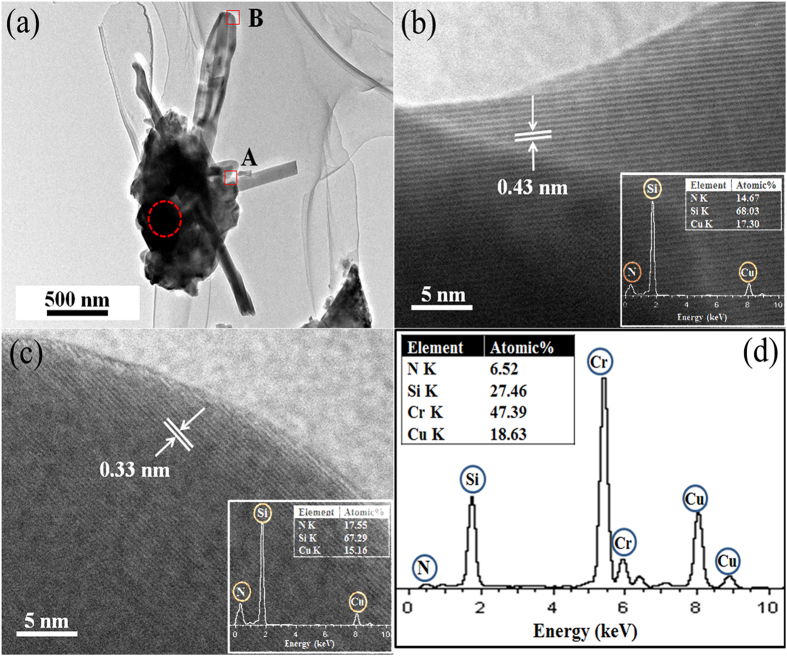
TEM images of the sample containing 10 wt% Cr: (**a**) A low-magnification TEM image of 1-D α-Si_3_N_4_ nanostructures which had just started to grow, (**b**,**c**) HRTEM images and corresponding EDS spectra (inset) of the 1-D α-Si_3_N_4_ nanostructures, detected from the areas A and B respectively in Fig. 7a, and (**d**) EDS of the root of 1-D α-Si_3_N_4_ nanostructures (highlighted by the dotted ring in the Fig. 7a).

In order to illustrate the role of Cr in the formation of these nanostructure materials, TEM images of the fired sample containing 10 wt% Cr were also taken. As shown in Fig. 7a, two types of α-Si_3_N_4_ nanostructures, i.e. nanowire with 100 nm in diameter and nanobelt with 400 nm in width, simultaneously grew from a particle. EDS analysis (insets in Fig. 7b,c) confirmed that the main parts and tips of the 1-D nanostructures contained only Si and N, but no Cr. Interestingly, Cr element was only detected at the root of the 1-D nanostructures (by EDS, Fig. 7d), which is believed to have acted as a “catalyst center” for the nucleation of the 1-D α-Si_3_N_4_ nanostructures.

### Mechanism of Cr catalyzed nitridation

The results presented and discussed above (Figs 1~7) suggested that Cr had played significant roles in accelerating the Si nitridation. To assist clarifying these roles, DFT calculations at the GGA-PBE/USP level of theory were carried out to simulate the adsorption behavior of a N_2_ molecule onto the Cr (001) surface and study the catalytic mechanism of Cr catalyst for nitridation reaction. The adsorption energy (E_ad_) of N_2_ on the para-position (90.24 kJ/mol) of the Cr (001) surface is higher than that on the ortho-position (66.24 kJ/mol) (Table 1), suggesting that adsorption of N_2_ onto the former is more favorable than the latter. Moreover, the bond lengths in a N_2_ molecule adsorbed on the para- and ortho-positions of Cr (001) surface are respectively 1.176 and 1.195 Å, which are longer than the original bond length (1.158 Å) in a free-standing N_2_ molecule (Table 1). Such increase in the bond length of N_2_, is believed to have assisted the dissociation of N_2_, as discussed previously^21^.

**Figure 8:**
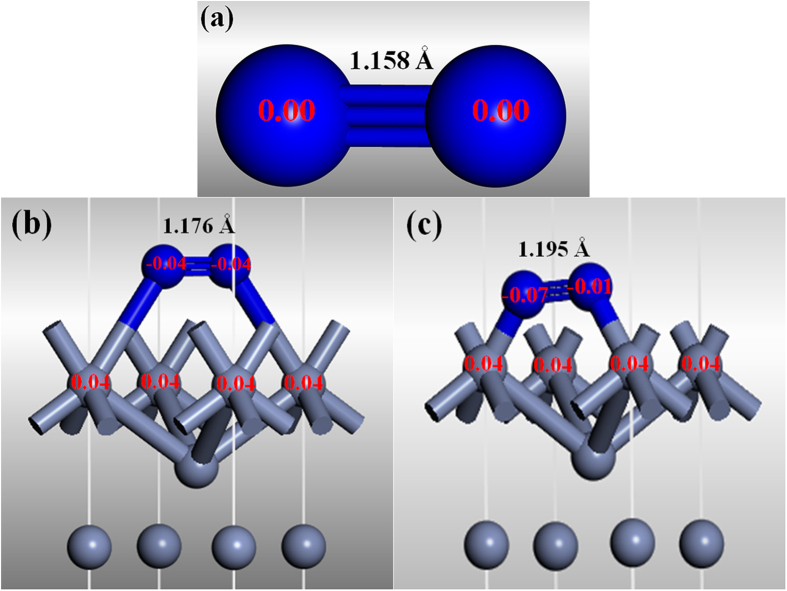
(**a**) Electronic structures of a free-standing N_2_ molecule. Adsorbed N_2_ on the para-position (**b**) and ortho-position (**c**) of Cr (001) surface.

The Mulliken atomic charge distributions of N and Cr atoms (Fig. 8 and Table 1) reveal that the N atoms are indeed negatively charged, whereas the Cr atoms are positively charged, providing further evidences on the electronic charge transfer from the latter to the former. In the case of para-position absorption, the two N atoms in a N_2_ molecule possess identical electronic charge. Moreover, the two N atoms absorbed on the ortho-position of Cr (001) surface have negligibly different negative-charges, due to the asymmetrical surrounding of the two N atoms, as also reported in our previous paper^21^. Since the Π_2py_* molecular orbital is an anti-bonding level, the bonding strength in a N_2_ molecule will be weaken if the adsorbed N_2_ molecule receives an electron from a Cr atom in the p-state. Changes in both bond length and Mulliken charge suggest that the N≡N bond can be weakened and the relatively stable N_2_ molecule activated when it is absorbed onto the surface of Cr.

## Discussion

**Figure 9:**
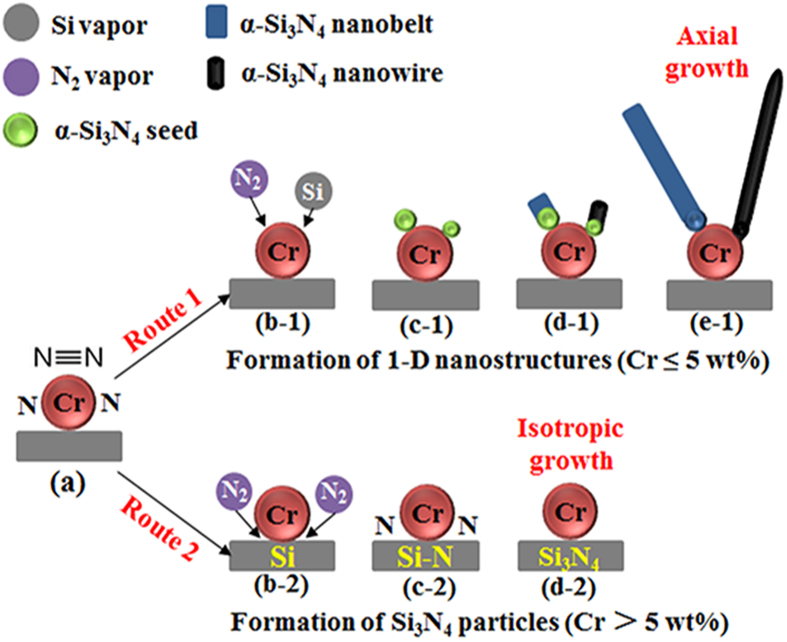
Schematic of proposed grow mechanism for 1-D α-Si_3_N_4_ nanostructures and Si_3_N_4_ particles for the samples with various amount of Cr.

As well documented in the literature^6^,^14^,^19^, the catalytic growth of 1-D nanostructure is generally controlled by the well-established VLS mechanism. The presence of a catalyst particle at the tip of a nanowire/nanorod/nanotube is often regarded as one of the main supportive evidences for this mechanism^29^. In the present work, Cr was found at the roots of the 1-D α-Si_3_N_4_ nanostructures, suggesting that Cr had played dominant roles in the nucleation of α-Si_3_N_4_ and the subsequent growth of the 1-D nanowires and nanobelts (Fig. 4). The detailed mechanisms can be schematically illustrated in Fig. 9. For the case of 1-D α-Si_3_N_4_ nanostructures, in the initial stage, N_2_ molecules diffused onto the surfaces of Cr particles (Fig. 9a). As predicted by the first-principle calculations (Section 3.2), the bond length in the N_2_ molecules would be increased and the bond strength decreased after their adsorption onto the Cr particles (see Fig. 8), resulting in activated N_2_ molecules. Subsequently, such N_2_ molecules would react with Si vapor generated from Reaction (1) (Fig. 9b-1), forming Si_3_N_4_ which would nucleate forming “crystal seeds” (Reaction (2)) on the surface of Cr (Fig. 9c-1). Owing to the hexagonal structure of Si_3_N_4_ (i.e., cell parameters a = b ≠ c), its different planes exhibit different surface energy values (J/m^−2^), for example, E(110): 1.95; E(010): 2.57; E(001): 2.74; and E(101): 2.77^30^. In this case, crystal surfaces with lower energies tend to serve as the enclosure surfaces, so the incoming Si and N preferred to diffuse to and deposit on the high energy surfaces (001) and (101) in the length directions [001] and [101], respectively, resulting in simultaneous formation of nanowires and nanobelts (Fig. 9d-1, Figs 5 and 6). Considering that the residual Cr catalyst was only detected in the roots of the nanostructures (Fig. 7) rather than at their tips, the VS mechanism should have dominated the growth process of the 1-D nanostructures.

Nevertheless, upon addition of >5% Cr, lots of Si_3_N_4_ particles rather than Si_3_N_4_ nanowires/nanobelts were formed in the samples (Figs 2 and 3), which can be explained by the following two reasons: 1) the formation of lots of Cr_x_Si_y_ alloy in the sample (Fig. 2) delayed the growth of 1-D α-Si_3_N_4_ (Fig. 3d) as a result of the low activation energy of nitrogen diffusion through the alloy^31^, and 2) when the N_2_ molecules diffused onto the Cr-Si interface (Fig. 9b-2), they would be activated by Cr forming active nitrogen species, thus accelerating the nitrdation rate of Si. With increasing the Cr addition, more activated N_2_ molecules would be generated at the interface (Fig. 9c-2). Consequently, the nitridation of Si would be promoted via a gas-solid reaction process (Reaction (3)), and more Si_3_N_4_ particles generated via isotropic formation from Si surface to the center (Fig. 9d-2).


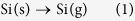



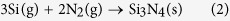



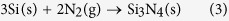


In summary, Cr exhibited a strong accelerating effect on the conversation of Si to Si_3_N_4_. At 1350 °C, the complete conversion of Si to Si_3_N_4_ was achieved in the samples containing 1.25 wt% Cr. When the Cr addition was 5 wt%, the catalyst efficiently promoted the formation of 1-D α-Si_3_N_4_ nanostructures. Si_3_N_4_ nanowires about 50 μm long and 50–200 nm in diameter, and nanobelts about 10 μm long and 300–1000 nm in width, were simultaneously obtained. The 1-D α-Si_3_N_4_ nanostructures grew from their Cr-containing roots via a VS mechanism. The accelerating effect of Cr on the nitridation of Si powder can be ascribed to the electron transfer from Cr to N, increasing the bond length and weakening bond strength in N_2_ molecules, as predicted by the first-principle calculations.

## Methods

### Raw materials and sample preparation

Si (99.9% pure, ≤44 μm, Naiou Nano Technology Co., Ltd., Shanghai, China) and Cr powders (≤6 μm, 99.9% pure, Naiou Nano Technology Co., Ltd., Shanghai, China) were used as the main starting materials, and high purity N_2_ (purity >99.999%) was used as the nitrogen source. Si powders were mixed with various amounts of Cr (0–10 wt%) for 30 min in a ball mill at 300 rpm. The mixed batch was pressed under 30 MPa forming cylindrical samples with 20 mm in height and 20 mm in diameter. The samples were placed in an alumina-tube furnace and fired at 1200–1350 °C for 3 h in flowing N_2_. As the nitridation reaction of Si is strongly exothermic, to avoid overheating induced Si melting and its negative effect in nitridation, the furnace was initially heated to 1150 °C and then 1280 °C and held at each of these temperatures for 1 h, before being further heated to the final target nitridation temperature.

### Sample characterization

Phase compositions in reacted samples were determined by X-ray diffraction (XRD, X’Pert Pro, Philips, Netherland). Spectra were recorded at 40 k*V* and 40 m*A* using Cu-*Kα* radiation, with a scanning rate of 2° (2*θ*)/min and a step size of 0.02° (2*θ*). ICDD cards used to identify Si, α-Si_3_N_4_ and β-Si_3_N_4_ are No. 01-089-5012, 01-073-1210 and 01-082-0697, respectively. The Rietveld refinement method was used to calculate crystalline phase contents in the fired samples. The overall conversion (OC) from Si to Si_3_N_4_ was determined using the quantitative analysis values of the Si, α- and β-Si_3_N_4_, and α-phase contents in final products were calculated based on the quantitative analysis values of the α- and β-Si_3_N_4_.

Microstructures and morphologies of the final products were observed by using a field emission scanning electron microscope (FESEM, Nova 400 Nano FESEM, FEI Co., USA, 15 k*V*) and a high-resolution transmission electron microscope (HRTEM, 2000 F, Jeol Ltd., Japan, 200 k*V*). The samples for SEM were coated with gold, and those for TEM were prepared by ultra-sonic dispersion of the sample powders in EtOH, followed by dropping and drying the suspension onto a copper grid, respectively. Selected area electron diffraction (SAED), along with an energy dispersive X-ray spectroscopy (EDS, Noran 623 M-3SUT, Thermo Electron Corporation, Japan) was used for assisting phase identifications.

### First-principle calculation

To assist understanding the catalytic nitridation mechanism in the case of using Cr as a catalyst, first-principle calculations based on the Cr (001) slab model were carried out using the CASTEP program based on the plane-wave pseudopotential (PW-PP) approach^32^ A vacuum space of 10 Å was introduced to prevent interactions between slabs. The outmost six layers and absorbed N_2_ molecules were fully relaxed.

The electron-ion interactions were represented by ultrasoft pseudopotentials (USP)^33^, and the electron-electron interactions were calculated by the generalized gradient approximation (GGA) with the Perdew-Burke-Ernzerhof (PBE) exchange correlation functional^34^. The equilibrium geometries were obtained by performing geometry optimization using the Broyden-Fletcher-Goldfarb-Shannon (BFGS) minimization method^35^, the energy cutoff for the plane wave basis set was set at 450 e*V*, and the Brillouin zone was sampled at 7 × 7 × 1 Monkhorst-Pack k-points. A Fermi smearing of 0.1 e*V* was utilized to speed up SCF convergence. The applied convergence criteria for geometry optimization were respectively 2.0×10^−5^ e*V*/atom, 0.05 e*V*/*Å* and 0.002 *Å* for energy, force and displacement.

## Additional Information

**How to cite this article**: Liang, F. *et al*. Catalytic Effects of Cr on Nitridation of Silicon and Formation of One-dimensional Silicon Nitride Nanostructure. *Sci. Rep.* **6**, 31559; doi: 10.1038/srep31559 (2016).

## Figures and Tables

**Table 1:** Electronic charge on N atoms, N-N bond length, and adsorption energy of N2 molecule on Cr (001) surface based on the first-principle calculations.

	Electronic charge	Bond length (Å)	E_ad_ (kJ/mol)
Free-standing N_2_ molecule	0.00, 0.00	1.158	—
N_2_ adsorbed on para-position Cr atoms	−0.04, −0.04	1.176	90.24
N_2_ adsorbed on ortho-position Cr atoms	−0.07, −0.01	1.195	66.24
